# Staging Parkinson's disease according to the MNCD classification correlates with caregiver burden

**DOI:** 10.1002/brb3.3295

**Published:** 2023-11-08

**Authors:** Diego Santos‐García, Teresa de Deus Fonticoba, Carlos Cores Bartolomé, María J. Feal Painceiras, Iago García Díaz, María Cristina Íñiguez Alvarado, Jose Manuel Paz, Silvia Jesús, Marina Cosgaya, Juan García Caldentey, Nuria Caballol, Ines Legarda, Jorge Hernández Vara, Iria Cabo, Lydia López Manzanares, Isabel González Aramburu, Maria A. Ávila Rivera, Víctor Gómez Mayordomo, Víctor Nogueira, Julio Dotor García‐Soto, Carmen Borrué, Berta Solano Vila, María Álvarez Sauco, Lydia Vela, Sonia Escalante, Esther Cubo, Zebenzui Mendoza, Juan C. Martínez Castrillo, Pilar Sánchez Alonso, Maria G. Alonso Losada, Nuria López Ariztegui, Itziar Gastón, Jaime Kulisevsky, Manuel Seijo, Caridad Valero, Ruben Alonso Redondo, Maria Teresa Buongiorno, Carlos Ordás, Manuel Menéndez‐González, Darrian McAfee, Pablo Martinez‐Martin, Pablo Mir, COPPADIS Study Group

**Affiliations:** ^1^ Department of Neurology, CHUAC Complejo Hospitalario Universitario de A Coruña A Coruña Spain; ^2^ Department of Neurology, CHUF Complejo Hospitalario Universitario de Ferrol A Coruña Spain; ^3^ Department of Neurology, Unidad de Trastornos del Movimiento, Servicio de Neurología y Neurofisiología Clínica, Instituto de Biomedicina de Sevilla Hospital Universitario Virgen del Rocío/CSIC/Universidad de Sevilla Seville Spain; ^4^ CIBERNED (Centro de Investigación Biomédica en Red Enfermedades Neurodegenerativas) Madrid Spain; ^5^ Department of Neurology Hospital Clínic de Barcelona Barcelona Spain; ^6^ Department of Neurology Centro Neurológico Oms 42 Palma de Mallorca Spain; ^7^ Department of Neurology, Consorci Sanitari Integral Hospital Moisés Broggi Sant Joan Despí Barcelona Spain; ^8^ Department of Neurology Hospital Universitario Son Espases Palma de Mallorca Spain; ^9^ Department of Neurology Hospital Universitario Vall d´Hebron Barcelona Spain; ^10^ Department of Neurology Complejo Hospitalario Universitario de Pontevedra (CHOP) Pontevedra Spain; ^11^ Department of Neurology Hospital Universitario La Princesa Madrid Spain; ^12^ Department of Neurology Hospital Universitario Marqués de Valdecilla – IDIVAL Santander Spain; ^13^ Department of Neurology, Consorci Sanitari Integral Hospital General de L´Hospitalet, L´Hospitalet de Llobregat Barcelona Spain; ^14^ Department of Neurology, Institute of Neuroscience Vithas Madrid La Milagrosa University Hospital, Vithas Hospital Group Madrid Spain; ^15^ Department of Neurology Hospital Universitario Lucus Augusti Lugo Spain; ^16^ Department of Neurology Hospital Universitario Virgen Macarena Sevilla Spain; ^17^ Department of Neurology Hospital Infanta Sofía Madrid Spain; ^18^ Department of Neurology Institut d'Assistència Sanitària (IAS) – Institut Català de la Salut Girona Spain; ^19^ Department of Neurology Hospital General Universitario de Elche Elche Spain; ^20^ Department of Neurology Fundación Hospital de Alcorcón Madrid Spain; ^21^ Department of Neurology Hospital de Tortosa Verge de la Cinta (HTVC) Tortosa Tarragona Spain; ^22^ Department of Neurology Complejo Asistencial Universitario de Burgos Burgos Spain; ^23^ Department of Neurology Hospital Universitario de Canarias San Cristóbal de la Laguna Santa Cruz de Tenerife Spain; ^24^ Department of Neurology Hospital Universitario Ramón y Cajal, IRYCIS Madrid Spain; ^25^ Department of Neurology Hospital Universitario Puerta de Hierro Madrid Spain; ^26^ Department of Neurology Hospital Álvaro Cunqueiro, Complejo Hospitalario Universitario de Vigo (CHUVI) Vigo Spain; ^27^ Department of Neurology Complejo Hospitalario de Toledo Toledo Spain; ^28^ Department of Neurology Complejo Hospitalario de Navarra Pamplona Spain; ^29^ Department of Neurology Hospital de Sant Pau Barcelona Spain; ^30^ Department of Neurology Hospital Arnau de Vilanova Valencia Spain; ^31^ Department of Neurology Hospital Universitari Mutua de Terrassa Terrassa Barcelona Spain; ^32^ Department of Neurology Hospital Rey Juan Carlos Madrid Spain; ^33^ Department of Neurology Hospital Universitario Central de Asturias Oviedo Spain; ^34^ University of Maryland School of Medicine Baltimore Maryland USA

**Keywords:** burden, caregiver, non‐motor symptoms, Parkinson's disease, stage

## Abstract

**Background and objective:**

Recently, we demonstrated that staging Parkinson's disease (PD) with a novel simple classification called MNCD, based on four axes (motor, non‐motor, cognition, and dependency) and five stages, correlated with disease severity and patients’ quality of life. Here, we analyzed the correlation of MNCD staging with PD caregiver's status.

**Patients and methods:**

Data from the baseline visit of PD patients and their principal caregiver recruited from 35 centers in Spain from the COPPADIS cohort from January 2016 to November 2017 were used to apply the MNCD total score (from 0 to 12) and MNCD stages (from 1 to 5) in this cross‐sectional analysis. Caregivers completed the Zarit Caregiver Burden Inventory (ZCBI), Caregiver Strain Index (CSI), Beck Depression Inventory‐II (BDI‐II), PQ‐10, and EUROHIS‐QOL 8‐item index (EUROHIS‐QOL8).

**Results:**

Two hundred and twenty‐four PD patients (63 ± 9.6 years old; 61.2% males) and their caregivers (58.5 ± 12.1 years old; 67.9% females) were included. The frequency of MNCD stages was 1, 7.6%; 2, 58.9%; 3, 31.3%; and 4–5, 2.2%. A more advanced MNCD stage was associated with a higher score on the ZCBI (*p* < .0001) and CSI (*p* < .0001), and a lower score on the PQ‐10 (*p* = .001), but no significant differences were observed in the BDI‐II (*p* = .310) and EUROHIS‐QOL8 (*p* = .133). Moderate correlations were observed between the MNCD total score and the ZCBI (*r* = .496; *p* < .0001), CSI (*r* = .433; *p* < .0001), and BDI‐II (*r* = .306; *p* < .0001) in caregivers.

**Conclusion:**

Staging PD according to the MNCD classification is correlated with caregivers’ strain and burden.

## Graphical abstract

Recently, it has been reported that staging Parkinson's disease (PD) with a novel simple classification called MNCD, based on 4 axes (Motor; Non‐motor; Cognition; Dependency) and 5 stages, correlated with disease severity and patients' quality of life. In this new manuscript, we observed that staging PD according to the MNCD classification correlated with caregivers' strain and burden.

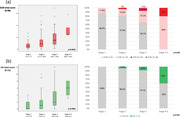

## 1 INTRODUCTION

Parkinson's disease (PD) is a complex and very heterogeneous progressive neurodegenerative disorder causing not only motor but also non‐motor symptoms (NMSs) that result in loss of patient autonomy for activities of daily living (ADL) and worse quality of life (QoL) impairment, but second, caregiver's strain and burden as well (Greenland et al., 2019; Mosley et al., 2017; Zhao et al., 2021). Moreover, caregiver's status impacts on patient's status as well, which has been named the “vicious circle of illness” (Santos‐García et al., 2023b). From a clinical point of view and with the aim of defining exhaustively the status of a patient with PD at each moment, a novel simple classification called “MNCD” has been proposed (Santos‐García et al., 2021a). The MNCD is based on four major axes (M, motor; N, non‐motor; C, cognition; D, dependency) and proposes five stages, from MNCD stage 1, no relevant symptoms, to MNCD stage 5, dementia and dependency for basic ADL, and a total score from 0 (0 + 0 + 0 + 0 = 0; the best possible status) to 12 (4 + 4 + 2 + 2 = 12; the worst possible status). Recently, we demonstrated using data from the COPPADIS cohort (Santos‐García et al., 2016, 2019) that staging PD with the MNCD classification correlated with disease severity and patients’ QoL (Santos‐García et al., 2023a). Based on these findings and awaiting the results of an ongoing validation study of the MNCD classification and of its application in a prospective follow‐up cohort, early observations suggest that the MNCD classification could be a useful tool to apply in PD patients to identify the main symptoms of the disease and to monitor the progression of the disorder. Neuropsychiatric symptoms, such as visual hallucinations, depression, cognitive impairment and/or dementia, falls, disability, and a more advanced stage disease, have been identified as patient factors associated with caregiver burden in PD (Aamodt et al., 2023). All these data are collected in the MNCD classification with also a proposed staging and scoring that indicates the degree of severity, from least to most. That is why we think that the MNCD classification can quickly provide us with information about the patient's condition that affects his/her QoL as well as the status of his/her principal caregiver. Importantly, with the aim to stop the vicious circle of illness in PD (Santos‐García et al., 2023b), the detection of burden in the principal caregiver of the patient is important and should be acted on as earlier as possible.

The aim of this new analysis was to study the correlation of the MNCD staging with PD caregiver's status. Data from the COPPADIS cohort (Santos‐García et al., 2021a) used in the previous publication (Santos‐García et al., 2023a) were used in this analysis. Our hypothesis was that caregivers’ status would be different between the different PD stages according to the MNCD classification, with a better QoL and mood when the patient has a low stage with a low score and vice versa, a worse mood and QoL with greater burden and stress when the patient has a higher stage and score (i.e., a more advanced MNCD stage, a worse caregiver's status).

## 2 MATERIALS AND METHODS

Data collected at the baseline visit from PD patients and their principal caregiver recruited from 35 hospitals in Spain from the COPPADIS cohort (Santos‐García et al., 2016) from January 2016 to November 2017 were used in this cross‐sectional transversal study. Methodology about COPPADIS‐2015 study has been published and can be consulted (Santos‐García et al., 2019). All patients included were diagnosed according to UK PD Brain Bank criteria (Hughes et al., 1992). Exclusion criteria were: non‐PD parkinsonism; a mini‐mental state examination <26; age <18 or >75 years; inability to read or understand the questionnaires; to be receiving any advanced therapy (continuous infusion of levodopa or apomorphine, and/or with deep brain stimulation); and the presence of comorbidity, sequelae, or any disorder that could interfere with the assessment.

### 2.1 PD patient assessment

Information on sociodemographic aspects, factors related to PD, comorbidity, and treatment, including levodopa equivalent daily dose (Schade et al., 2020), were collected at baseline. As this analysis was focused on the caregiver status and the results about the relationship between the MNCD staging and patients’ symptoms have been already published (Santos‐García et al., 2023a), all the information collected about motor, NMS, QoL, and autonomy for ADL in PD patients (Santos‐García et al., 2019) were not provided on this occasion.

### 2.2 Caregiver assessment

A person who, without being a professional and/or receiving money in exchange for services, lived with the patient and was responsible for his/her was included as a primary caregiver (Santos‐García et al., 2016). His/her participation was voluntary, and a written informed consent was collected for all participants. In caregivers, four aspects were analyzed using validated specific scales: burden (Zarit Caregiver Burden Inventory [ZCBI]), strain (Caregiver Strain Index [CSI]), mood (Beck Depression Inventory‐II [BDI‐II]), and global QoL (PQ‐10 and EUROHIS‐QOL 8‐item index [EUROHIS‐QOL8]). The ZCBI (Novak & Guest, 1989) contains 22 items that rate the impact of the disease on the caregiver's physical, emotional, and socioeconomic status. Responses are scored on a scale from 0 (never) to 4 (nearly always). The maximum total score, indicative of the highest burden, is 88. The CSI (Robinson, 1983) is a 13‐item questionnaire designed to assess the level of stress experienced by caregivers. There are two possible responses for each item: “yes” or “no”. The total score is the result of adding all positive responses (from 0, no stress, to 13, maximum level of stress). Mood was assessed with the BDI‐II (Beck et al., 1996). This is a self‐administered, 21‐item instrument. It has been designed to assess the severity of depression symptoms in adults and adolescents with a minimum age of 13 years. The evaluated subject must choose one of four alternatives (ordered from lesser to greater severity), in each item, which best describes his/her status over the previous 2 weeks. The score ranges from 0 (minimum) to 63 (maximum). Higher scores will reflect, a priori, a worse mood. Finally, global QoL was measured with the PQ‐10 and the EUROHIS‐QOL8. The PQ‐10 (Santos‐García et al., 2019) is a rating of global perceived QoL based on a scale from 0 (worst) to 10 (best). The EUROHIS‐QOL8 (Da Rocha et al., 2012) is an 8‐item QoL questionnaire (QoL; health status; energy; autonomy in ADL; self‐esteem; social relationships; economic capacity; habitat) derived from the WHOQOL‐100 and the WHOQOL‐BREF. For each item, the score ranges from 0 (not at all) to 5 (completely). The total score is expressed as the mean of the individual scores. A higher score indicates a higher QoL. Sociodemographic data about the principal caregiver of the PD patient was also collected.

### 2.3 MNCD classification

The MNCD classification was applied using the same criteria recently reported in the COPPADIS cohort (Santos‐García et al., 2023). This new classification (Santos‐García et al., 2021a) is based on four axes: (1) motor symptoms, (2) NMSs, (3) cognition, and (4) dependency for ADL. The first axis (motor symptoms) is subdivided into four defined sub‐axes: (1) motor fluctuations, (2) dyskinesia, (3) axial symptoms, and (4) tremor. The second axis (NMSs) is subdivided into four defined sub‐axes: (1) neuropsychiatric symptoms, (2) autonomic dysfunction, (3) sleep disturbances and fatigue, and (4) pain and sensory disorders. Regarding the third axis (cognition), patients are classified as having normal cognition, with mild cognitive impairment or dementia. Finally, patients are classified according to the fourth axis (dependency) as having independency for ADL, with dependency for instrumental or with dependency for basic activities. Patients were classified in four groups according to the MNCD stage: stage 1 (the patient has no relevant symptoms); stage 2 (there is at least one motor symptom or one NMS scoring in the MNCD classification); stage 3 (there is mild cognitive impairment and/or dependency for instrumental ADL); stages 4–5 (there is dementia and/or dependency for basic ADL). Moreover, the MNCD total score (from 0 to 12) was calculated according to the sum of the score of all axes of the MNCD classification: M (from 0 to 4) + N (from 0 to 4) + C (from 0 to 2) + D (from 0 to 2).

### 2.4 Data analysis

Data were processed using SPSS 20.0 for Windows. Only PD patients and their caregivers from the COPPADIS cohort with data collected about the MNCD classification and the ZCBI, CSI, BDI‐II, PQ‐10, and EUROHIS‐QOL8, respectively, were included in the analysis. ZCBI and CSI scores were expressed as quantitative and qualitative variables. Relative to the ZCBI score (from 0 to 88), four groups were defined (Santos‐García & Añón, 2012; Santos‐García & de la Fuente‐Fernández, 2015): little or no burden (0–20); mild‐to‐moderate burden (21–40); moderate‐to‐severe burden (41–60); severe burden (61–88). Two groups were considered according to CSI score (from 0 to 13) (Santos‐García & Añón, 2012; Santos‐García & de la Fuente‐Fernández, 2015): no high level of stress (0–6); high level of stress (7–13). With regard to mood (assessed by BDI‐II]), caregivers were classified as with major depression, minor depression, subthreshold depression, or non‐depression (Santos‐García et al., 2019). Specifically, regarding items 1, 4, 5, 9, 13, 15, 16, 17, and 18 of the BDI‐II, depression was defined: major depression, ≥5 symptoms with the presence of item 1 (feeling of sadness) and/or item 4 (anhedonia) (DSM‐IV criteria); minor depression, from 2 to 4 symptoms with the presence of item 1 and/or item 4 (DSM‐IV criteria); subthreshold depression, from 2 to 4 symptoms without the presence of item 1 and item 4 (Judd criteria) (American Psychiatric Association, 1994; Judd et al., 1994).

For comparison of caregivers’ burden (ZCBI), strain (CSI), mood (BDI‐II), and QoL (PQ‐10 and EUROHIS‐QOL8) between patients with a different MNCD stage (all stages together or two consecutive stages), the Student *t*‐test, the Mann–Whitney *U* test, Chi‐square test, Fisher test, ANOVA test, or the Kruskal–Wallis test were used as appropriate (distribution for variables was verified by one‐sample Kolmogorov–Smirnov test). Spearman's or Pearson's correlation coefficient, as appropriate, was used for analyzing the relationship between the MNCD total score (from 0 to 12) and the total score on ZCBI, CSI, BDI‐II, PQ‐10, and EUROHIS‐QOL8 on caregivers. Correlations were considered weak for coefficient values ≤.29, moderate for values between .30 and .59, and strong for values ≥.60. Linear regression models were used to determine in what grade the MNCD stage and the MNCD total score were associated with caregivers’ burden (ZCBI), strain (CSI), mood (BDI‐II), and QoL (PQ‐10 and EUROHIS‐QOL8). Age, gender, disease duration (years from symptoms onset), and caregiver's sociodemographic variables (age, gender, relationship with the patient, civil status, habitat, time as a caregiver, living with the patient or not, full‐time care or not and care of others or not) were included as covariates. With the aim of having a very strong evidence against the null hypothesis, the *p*‐value was considered significant (highly significant) when it was ≤.001 (Krikwood & Sterne, 2003).

### 2.5 Standard protocol approvals, registrations, and patient consents

Approval from the *Comité de Ética de la Investigación Clínica de Galicia* (2014/534; 02/DEC/2014) and a written informed consent from all participants in this study were obtained. COPPADIS‐2015 was classified by the AEMPS (*Agencia Española del Medicamento y Productos Sanitarios*) as a post‐authorization prospective follow‐up study with the code COH‐PAK‐2014‐01.

#### 2.5.1 Data availability

The protocol and the statistical analysis plan are available on request. Deidentified participant data are not available for legal and ethical reasons.

## 3 RESULTS

Two hundred and twenty‐four PD patients (63 ± 9.6 years old; 61.2% males) and their caregivers (58.5 ± 12.1 years old; 67.9% females) were included. The mean disease duration was 5.6 ± 4.1 years. Regarding MNCD stages, the distribution was stage 1, 7.6% (*N* = 17); stage 2, 58.9% (*N* = 132); stage 3, 31.3% (*N* = 70); stages 4–5, 2.2% (*N* = 5; four patients in stage 4 and only one patient in stage 5 from the MNCD classification according to the original description [Santos‐García et al., 2021a]). Nearly 70% of the caregivers were females (67.9%) and up to 92.4% were living with the patient and 21.9% taking care of the patient full time (33.5% of the patients had cognitive impairment and/or needed some help for some ADL). No significant differences were observed in sociodemographic aspects between caregivers of PD patients in the different stages except in age (*p* = .002), being younger the caregivers of those patients in stages 4–5 (in this group, 40% of the caregivers were the son or the daughter) (Table 1).

**TABLE 1:** Sociodemographic details and data about the status of the principal caregiver of patients with Parkinson's disease (PD) according to the motor, non‐motor, cognition, and dependency (MNCD) stage (*N* = 224).

	Stage 1 (*N* = 17; 7.6%)	Stage 2 (*N* = 132; 58.9%)	Stage 3 (*N* = 70; 31.3%)	Stages 4–5 (*N* = 5; 2.2%)	Total (*N* = 224)	*p*
**Patients**						
Age	63.2 ± 8.3	60.2 ± 10.2	68.3 ± 6.6	63.8 ± 8.3	63 ± 9.6	<.0001
Males (%)	64.7	61.4	58.6	80	61.2	.855
Disease duration (years)	3.4 ± 1.9	5.4 ± 4.1	6.3 ± 4.4	7.5 ± 1.9	5.6 ± 4.2	.062
l‐Dopa eq. daily dose (mg)	303.4 ± 255.1	541.9 ± 391.9	666.2 ± 427.9	1218 ± 775.9	574.8 ± 420.9	<.0001
Number of non antip. drugs	3 [0, 4]	2 [1, 4]	3 [2, 6.25]	5.5 [1.25, 6]	2 [1, 5]	<.0001
Hoehn & Yahr	2 [1.5, 2]	2 [1.5, 2]	2 [2, 2.5]	3.5 [2.25, 4]	2 [2, 2]	<.0001
MNCD—total score	0	2.6 ± 1.5	4.5 ± 1.9	7.6 ± 1.3	3.1 ± 2.1	<.0001
**Caregivers**						
Age	60.7 ± 10.1	56.4 ± 11.5	62.5 ± 11.6	49.8 ± 9.1	58.5 ± 12.1	.002
Females (%)	76.5	68.2	64.3	80	67.9	.761
Relationship (%):						.191
Spouse	94.1	87.1	82.9	60	85.7	
Son or daughter	5.9	7.6	14.3	40	10.3	
Other	0	5.3	2.6	0	4	
Civil status (%):						.938
Married	88.2	86.4	88.6	100	87.5	
Single	5.9	6.8	8.6	0	7.1	
Other	5.9	6.8	2.8	0	5.6	
Habitat (%):						.911
Rural	17.6	13.6	12.9	20	13.8	
Semiurban	29.4	22	20	20	21.9	
Urban	52.9	64.4	67.1	60	64.3	
Time as a caregiver (%):						.135
<1 year	29.4	12.1	8.6	0	12.1	
1–5 years	35.3	44.7	30	20	38.8	
>5, <10 years	17.6	19.7	24.3	20	21.9	
≥10 years	17.6	23.5	37.1	60	27.2	
Living with the patient (%)	100	93.2	90	80	92.4	.283
Full time care (%)	11.8	20	25.7	40	21.9	.411
Care of others (%)	52.9	34.8	27.1	60	34.4	.111
ZCBI	7.4 ± 7.7	11.7 ± 10.6	18.1 ± 14.2	33.6 ± 14.5	13.9 ± 12.6	<.0001
Moderate‐to‐severe burden (%)	0	3	7.1	20	4.4	.001
CSI	1.1 ± 1.5	1.6 ± 2.1	2.8 ± 2.8	5.8 ± 2.3	2 ± 2.5	<.0001
High level of stress (%)	0	3.8	8.6	40	5.8	.022
BDI‐II	4.4 ± 3.7	7.1 ± 8.5	8.2 ± 8.1	9.8 ± 5.7	7.3 ± 8.1	.31
Depressive symptoms (%):	35.3	40.8	47.1	60		.344
Major depression	5.9	13.6	18.6	20		
Minor depression	23.5	13.6	21.4	40		
Subthreshold depression	5.9	13.6	7.1	0		
EUROHIS‐QOL8	4.1 ± 0.5	3.9 ± 0.6	3.8 ± 0.5	3.6 ± 0.6	3.9 ± 0.6	.133
Quality of life	4.2 ± 0.8	3.9 ± 0.8	3.6 ± 0.8	2.8 ± 1.3	3.8 ± 0.8	.001
Health status	3.7 ± 0.9	3.7 ± 0.9	3.7 ± 0.8	3.6 ± 1.1	3.7 ± 0.9	.968
Energy	4 ± 0.9	3.9 ± 0.9	3.8 ± 0.8	3.4 ± 1.1	3.9 ± 0.9	.357
Autonomy for ADL	4.1 ± 0.8	4 ± 0.8	4 ± 0.9	3.8 ± 0.8	4 ± 0.8	.917
Self‐esteem	4.2 ± 0.4	3.9 ± 0.7	3.9 ± 0.6	3.4 ± 0.5	3.9 ± 0.7	.064
Social relationships	4.2 ± 0.5	4 ± 0.7	4 ± 0.7	3.9 ± 0.7	4 ± 0.7	.529
Economic capacity	4.2 ± 0.7	3.9 ± 0.8	3.6 ± 0.8	3.4 ± 0.9	3.8 ± 08	.016
Habitat	4.4 ± 0.7	4.3 ± 0.8	4.3 ± 0.5	4.2 ± 0.4	4.3 ± 0.7	.959
PQ‐10	8.4 ± 1.4	7.4 ± 1.4	7 ± 1.6	5.6 ± 2.5	7.3 ± 1.6	.001

*Note*: The results represent percentages, mean ± SD, or median [p25, p75]. Fisher exact test and ANOVA test were applied. Data about H&Y are during the OFF state (first thing in the morning without taking medication in the previous 12 h).

Abbreviations: ADL, activities of daily living scale; BDI‐II, Beck Depression Inventory‐II; EUROHIS‐QOL8, EUROHIS‐QOL 8‐item index; PD, Parkinson's disease.

**FIGURE 1:**
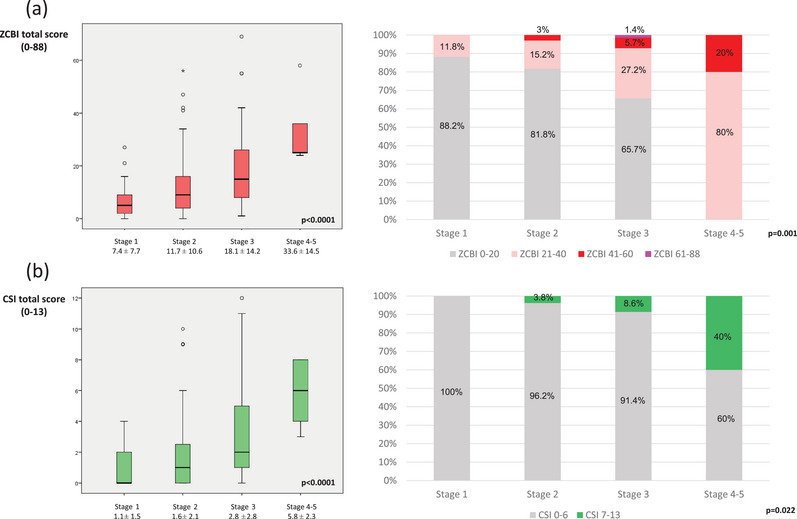
(a) Zarit Caregiver Burden Inventory (ZCBI) total score (on the left) and frequency of caregivers with different burden degree (on the right) regarding to the motor, non‐motor, cognition, and dependency (MNCD) stage, from stage 0 to stages 4–5. (b) Caregiver Strain Index (CSI) total score (on the left) and frequency of caregivers with high level (CSI > 6) versus no high level of stress (on the left) regarding to the MNCD stage, from stage 0 to stages 4–5.

A more advanced MNCD stage was associated with a higher score on the ZCBI (from 7.4 ± 7.7 at stage 1 to 33.6 ± 14.5 at stages 4–5; *p* < .0001) and CSI (from 1.1 ± 1.5 at stage 1 to 5.8 ± 2.3 at stages 4–5; *p* < .0001) (Table 1). One out of four caregivers (24.6%) had burden (mild‐to‐moderate, 20.1%; moderate‐to‐severe, 4%; severe burden 0.4%) and 5.8% high level of stress (CSI > 6). At stage 1, only 11.8% of the caregivers had burden (mild‐to‐severe; ZCBI 21‐88) compared to 18.2% at stage 2, 34.3% at stage 3, and 100% at stages 4–5 (*p* = .001) (Figure 1). The frequency of the high level of stress in the different groups was 0% at stage 1; 0% at stage 2; 8.6% at stage 3; 40% at stages 4–5 (*p* = .022) (Figure 1). Moderate correlations were observed between the MNCD total score and the ZCBI (*r* = .496; *p* < .0001) and between the MNCD total score and the CSI (*r* = .433; *p* < .0001).

**FIGURE 2:**
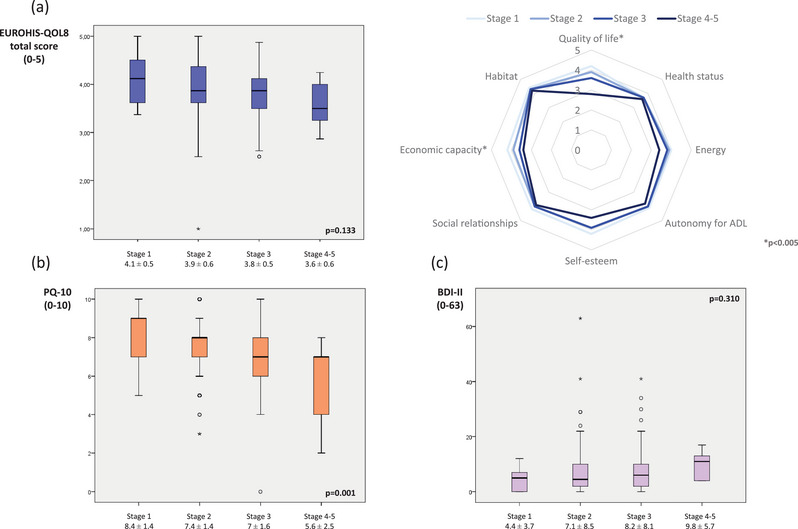
(a) EUROHIS‐QOL 8‐item index (EUROHIS‐QOL8) total score (on the left) and mean value of each domain of the scale (on the right) regarding to the motor, non‐motor, cognition, and dependency (MNCD) stage, from stage 0 to stages 4–5. (b) PQ‐10 score regarding to the MNCD stage, from stage 0 to stages 4–5. (c) Beck Depression Inventory II (BDI‐II) total score regarding to the MNCD stage, from stage 0 to stages 4–5.

Regarding mood, no significant differences were detected between different stages neither in the BDI‐II total score (*p* = .310) nor groups according to depressive symptoms (*p* = .330) (Table 1 and Figure 2). However, the correlation between the MNCD stage and the BDI‐II total score was moderate (*r* = .306; *p* < .0001). Moderate correlations were observed between the BDI‐II and the ZCBI (*r* = .541; *p* < .0001) and the BDI‐II and the CSI (*r* = .599; *p* < .0001). Global QoL was related to MNCD stage when the PQ‐10 was used, with a lower score at a higher stage (from 8.4 ± 1.4 at stage 1 to 5.6 ± 2.5 at stage 4; *p* = .001) (Table 1 and Figure 2). However, no significance was detected when the EUROHIS‐QOL8 was used (*p* = .133) (Table 1). By domains, significant differences were observed in “quality of life” and “economic capacity” between groups (from MNCD stage 1 to stages 4–5), with a lower score at a higher stage (Table 1 and Figure 2). Correlations between the MNCD total score and the PQ‐10 and between the MNCD total score and the EUROHIS‐QOL8 were weak (Table 2). As the proof of the utility of the PQ‐10, the correlation between the PQ‐10 and the EUROHIS‐QOL8 was strong (*r* = .735; *p* < .0001).

**TABLE 2:** Correlations between the motor, non‐motor, cognition, and dependency (MNCD) total score (from 0 to 12) and the other variables about the status of the caregiver (Zarit Caregiver Burden Inventory [ZCBI], Caregiver Strain Index [CSI], Beck Depression Inventory‐II [BDI‐II], PQ‐10, EUROHIS‐QOL 8‐item index [EUROHIS‐QOL8]).

	MNCD	ZCBI	CSI	BDI‐II	PQ‐10	EUROHIS‐QOL8
**MNCD**	N.A.	*r* = .496 *p* < .0001	*r* = .433 *p* < .0001	*r* = .306 *p* < .0001	*r* = −.238 *p* < .0001	*r* = −.193 *p* = .004
ZCBI	*r* = .496 *p* < .0001	N. A.	*r* = .651 *p* < .0001	*r* = .541 *p* < .0001	*r* = −.421 *p* < .0001	*r* = −.441 *p* < .0001
CSI	*r* = .433 *p* < .0001	*r* = .651 *p* < .0001	N. A.	*r* = .599 *p* < .0001	*r* = −.446 *p* < .0001	*r* = −.486 *p* < .0001
BDI‐II	*r* = .306 *p* < .0001	*r* = .651 *p* < .0001	*r* = .599 *p* < .0001	N. A.	*r* = −.497 *p* < .0001	*r* = −.557 *p* < .0001
PQ‐10	*r* = −.238 *p* < .0001	*r* = −.421 *p* < .0001	*r* = −.446 *p* < .0001	*r* = −.497 *p* < .0001	N. A	*r* = .704 *p* < .0001
EUROHIS‐QOL8	*r* = −.193 *p* = .004	*r* = −.441 *p* < .0001	*r* = −.486 *p* < .0001	*r* = −.557 *p* < .0001	*r* = .704 *p* < .0001	N. A.

*Note*: Spearman correlation test were applied. Correlations between all variables are also shown.

After controlling for covariates mentioned in methods (age; gender; disease duration; caregiver's sociodemographic variables), the MNCD stage was significantly associated to ZCBI (*β* = .36; 95%CI 4.42–10.25; *R* ^2^ = 0.34; *p* < .0001), CSI (*β* = .27; 95%CI .44–1.59; *R* ^2^ = .31; *p* = .001), and PQ‐10 (*β* = −.32; 95%CI −1.27 to −.39; *R* ^2^ = .12; *p* < .0001). Regarding the MNCD total score, the significance was observed to ZCBI (*β* = .36; 95%CI 1.14–2.98; *R* ^2^ = .32; *p* < .0001) and CSI (*β* = .3; 95%CI.15–0.51; *R* ^2^ = .25; *p* < .0001), with a trend of significance to BDI‐II (*β* = .23; 95%CI .25–1.6; *R* ^2^ = .17; *p* = .007), EUROHIS‐QOL8 (*β* = −.21; 95%CI −.11 to −.09; *R* ^2^ = .03; *p* = .024) and PQ‐10 (*β* = −.32; 95%CI −.37 – −.1; *R* ^2^ = .03; *p* = .01).

## 4 DISCUSSION

The present study observed that a more advanced stage in PD patients according to the MNCD classification was associated with greater strain and burden (ZCBI; CSI) and a worse QoL (PQ‐10) in the principal caregiver. This finding, combined with previous reports (Santos‐García et al., 2023), supports that the MNCD classification could be a useful tool to monitor the progression of PD.

PD is a complex and very heterogeneous disorder with a variable prognosis between patients. From a clinical point of view and looking at the disease as a whole, four cardinal aspects in the patient linked to outcome are motor symptoms, NMS, cognition, and the ability to perform day‐to‐day activities. Throughout the evolution of the disease, motor and NMS burden increase, and progressive cognitive impairment and dependency for ADL appear. However, many phenotypes in PD have been reported, and disability can appear very early in some patients or late in the disease course in others (Lawton et al., 2018; Fereshtehnejad et al., 2015; De Pablo‐Fernández et al., 2019). In this context, a classification to monitor PD is required. Although the Hoehn and Yahr (1967) stage is used very frequently in clinical practice because it is very simple, it provides information only about the motor stage. As NMS or cognitive dysfunction can be present at the first steps of the disease or even before motor symptoms development and they impact on QoL (Santos‐García et al., 2021c; Goldman & Postuma, 2014), they should be identified and monitored from the first moment (i.e., at diagnosis) (Zis et al., 2015). In fact, NMSs have gained in importance in the latest proposed diagnostic criteria of PD (Postuma et al., 2015; Berg et al., 2015; Heinzel et al., 2019). These four capitals’ aspects commented are the basement of a new proposed classification for PD called MNCD (Santos‐García et al., 2023). The MNCD classification includes four major axes (M, motor symptoms; N, NMS; C, cognition; D, dependency for ADL) and five stages with the aim to categorize and differentiate patients in a different evolutionary stage, being the first PD classification taking into account key aspects of the disease, such as axial symptoms, motor fluctuations, NMS, cognitive problems, and their impact on disability.

Although it is necessary to apply the MNCD in clinical practice and demonstrate good inter‐ and intra‐variability, a very recent application of this tool using retrospectively the database of the baseline visits from the Spanish Cohort COPPADIS found that the MNCD staging was associated with disease severity and patient's QoL (Santos‐García et al., 2023). That is, a more advanced MNCD stage corresponded to a greater disease severity in terms of motor symptoms and NMS and a worse QoL and autonomy for ADL. On the other hand, the MNCD total score (from 0 to 12) correlated strongly with health‐related (PDQ‐39) and global (EUROHIS‐QOL8) QoL in PD patients (Santos‐García et al., 2023). In this new analysis using the same database and with the aim of gathering more information about the potential role of the MNCD classification in PD, we wanted to know what the status of the principal caregiver of a PD patient with respect to the MNCD classification was. Many studies have identified symptoms related to caregiver burden, such as depression, visual hallucinations, sleep disturbances, cognitive impairment, falls, a more advanced stage disease, and a greater disability (Santos‐García & de la Fuente‐Fernández, 2015; Schrag et al., 2006; Martinez‐Martin et al., 2007; D'Amelio et al., 2009; Martinez Martin et al., 2012; Smith et al., 2019; Peters et al., 2011; Santos‐García et al., 2022). Based on this, our hypothesis was that caregivers’ status would be different between the different MNCD stages, with a better mood and QoL in stage 1 and a worse QoL and mood and a greater strain and burden in caregivers at a more advanced stage (i.e., a more advanced MNCD stage, a worse caregiver's status). We found differences for caregiver burden (ZCBI), strain (CSI), and QoL (PQ‐10) but not for mood (BDI‐II). All caregivers of PD patients in stages 4–5 had some degrees of burden compared to only 11.8% at stage 1. Additionally, up to 40% of caregivers of PD patients in stages 4–5 reported a high level of stress compared to none of the caregivers of stage 1 PD patients. However, it is important to be cautious because although the sample size was 224 caregivers, only 5 were of PD patients in the most advanced stage (stages 4 and 5). The very small sample size of the group with MNCD stages 4–5 (*N* = 5) suggests that these findings need to be replicated and are not entirely reliable. On the other hand, some of the non‐statistically significant differences (EUROHIS‐QOL8; BDI‐II) may be due to a small sample size (e.g., 60% of caregivers of patients at stages 4–5 had depressive symptoms compared to 35.3% at stage 1). Of note, a moderate correlation was observed between the caregiver's BDI‐II total score and the MNCD total score (from 0 to 12), and between mood and burden and mood and strain in the caregiver, suggesting the necessity of being alert about depressive symptoms due to its important implications (Santos‐García et al., 2023) in the principal caregiver of a patient with a greater disease burden according to the MNCD classification. An ideal scenario could be to apply the MNCD classification in clinical practice to the patient at the first visit (i.e., a naïve patient) and to monitor its changes in each visit in the short‐, medium‐, and long‐term (i.e., >15 years) in a big cohort of PD patients and caregivers (i.e., >500) in a prospective follow‐up study. As an alternative, applying the MNCD classification in a big cohort (i.e., >1,000) in only one visit (cross‐sectional study) with many PD patients in all stages (i.e., from 1 to 5 of Hoehn & Yahr, 1967) would avoid the frequent bias of not including very advanced PD patients (e.g., with dementia and institutionalized). Finally, and regarding QoL, the PQ‐10 was a good marker of global QoL related to the MNCD staging. It is a simple question about the global QoL perception (from 0, the worst, to 10, the best), and in‐line with many previous observations in the COPPADIS cohort (Santos‐García et al., 2019; Santos‐García et al., 2021b), we recommend using it as a very simple and quick marker of global QoL for PD patients and their caregivers as an alternative to others. Indeed, the correlation between the PQ‐10 and the EUROHIS‐QOL8 was strong (*r* > .7).

This study has important limitations; many of them commented in the recent report about the correlation between disease severity and QoL in PD patients from the COPPADIS cohort and the MNCD staging (Santos‐García et al., 2023): The MNCD classification was applied retrospectively and not directly applied by the neurologist during a face‐to‐face assessment; a bias toward less advanced PD in this cohort with only five caregivers of PD patients in stages 4–5; a cross‐sectional instead of a prospective follow‐up analysis; a study to demonstrate good inter‐ and intra‐variability is needed. On the contrary, the results of this study are again novel and support the idea to consider the MNCD classification as a simple tool to use in PD patients for identifying the main symptoms and monitor the progression of PD.

In conclusion, we observed in this study that staging PD according to the MNCD classification correlates with caregiver burden. More studies are needed to demonstrate the potential value of this new simple proposed tool. The backing of a scientific society may be key to its prosperous use by the scientific community.

## AUTHOR CONTRIBUTIONS

**Diego Santos‐García**: Conceptualization; investigation; writing—original draft; methodology; validation; software; formal analysis; data curation; supervision; resources. **Teresa de Deus Fonticoba**: Investigation; methodology; writing—review and editing. **Carlos Cores Bartolomé**: Investigation; writing—review and editing. **María J. Feal Painceiras**: Investigation; writing—review and editing. **Iago García Díaz**: Investigation; writing—review and editing. **María Cristina Íñiguez Alvarado**: Investigation; writing—review and editing. **Jose Manuel Paz**: Investigation; writing—review and editing. **Silvia Jesús**: Investigation; writing—review and editing; methodology. **Marina Cosgaya**: Investigation; writing—review and editing; methodology. **Juan García Caldentey**: Investigation; writing—review and editing; methodology. **Nuria Caballol**: Investigation; writing—review and editing; methodology. **Ines Legarda**: Investigation; writing—review and editing; methodology. **Jorge Hernández Vara**: Investigation; writing—review and editing; methodology. **Iria Cabo**: Investigation; writing—review and editing; methodology. **Lydia López Manzanares**: Investigation; writing—review and editing; methodology. **Isabel González Aramburu**: Investigation; writing—review and editing; methodology. **Maria A. Ávila Rivera**: Investigation; writing—review and editing; methodology. **Víctor Gómez Mayordomo**: Investigation; writing—review and editing; methodology. **Víctor Nogueira**: Investigation; writing—review and editing; methodology. **Julio Dotor García‐Soto**: Investigation; writing—review and editing; methodology. **Carmen Borrué**: Investigation; writing—review and editing; methodology. **Berta Solano Vila**: Investigation; writing—review and editing; methodology. **María Álvarez Sauco**: Investigation; methodology; writing—review and editing. **Lydia Vela**: Investigation; writing—review and editing; methodology. **Sonia Escalante**: Investigation; methodology; writing—review and editing. **Esther Cubo**: Investigation; writing—review and editing; methodology. **Zebenzui Mendoza**: Investigation; writing—review and editing; methodology. **Juan C. Martínez Castrillo**: Investigation; writing—review and editing; methodology. **Pilar Sánchez Alonso**: Investigation; writing—review and editing; methodology. **Maria G. Alonso Losada**: Investigation; methodology; writing—review and editing. **Nuria López Ariztegui**: Investigation; writing—review and editing; methodology. **Itziar Gastón**: Investigation; writing—review and editing; methodology. **Jaime Kulisevsky**: Investigation; writing—review and editing; methodology. **Manuel Seijo**: Investigation; writing—review and editing; methodology. **Caridad Valero**: Investigation; writing—review and editing; methodology. **Ruben Alonso Redondo**: Investigation; writing—review and editing; methodology. **Maria Teresa Buongiorno**: Investigation; writing—review and editing; methodology. **Carlos Ordás**: Investigation; writing—review and editing; methodology. **Manuel Menéndez‐González**: Investigation; writing—review and editing; methodology. **Darrian McAfee**: Writing—review and editing. **Pablo Martinez‐Martin**: Writing—review and editing; supervision. **Pablo Mir**: Investigation; conceptualization; writing—review and editing; methodology; supervision.

## CONFLICT OF INTEREST STATEMENT

Diego **Santos‐García** has received honoraria for educational presentations and advice service by Abbvie, UCB Pharma, Lundbeck, KRKA, Zambon, Bial, Italfarmaco, Teva, Archímedes, Esteve, Stada, Merz, and grants from the Spanish Ministry of Economy and Competitiveness [PI16/01575] co‐founded by ISCIII (Concesión de subvenciones de Proyectos de Investigación en Salud de la convocatoria 2020 de la Acción Estratégica en Salud 2017–2020 por el proyecto “PROGRESIÓN NO MOTORA E IMPACTO EN LA CALIDAD DE VIDA EN LA ENFERMEDAD DE PARKINSON”).

Teresa **de Deus Fonticoba**: None.

Carlos **Cores Bartolomé** has received honoraria for educational presentations and advice service by Lundbeck and UCB Pharma.

María J. **Feal Painceiras**: None.

Iago **García Díaz** has received support for educational activity from Bial.

María Cristina **Íñiguez Alvarado**: None.

Jose Manuel **Paz** has received honoraria/support for educational presentations and attending meetings from UCB, Abbvie, Zambon, Bial and KRKA.

Silvia **Jesús** has received honoraria from AbbVie, Bial, Merz, UCB, and Zambon and holds the competitive contract “Juan Rodés” supported by the Instituto de Salud Carlos III. She has received grants from the Spanish Ministry of Economy and Competitiveness (PI18/01898) and the Consejería de Salud de la Junta de Andalucía (PI‐0459‐2018).

Marina **Cosgaya**: None.

Juan **García Caldentey** has received honoraria for educational presentations and advice service by Qualigen, Nutricia, Abbvie, Italfarmaco, UCB Pharma, Lundbeck, Zambon, Bial, and Teva.

Nuria **Caballol** has received honoraria from Bial, Italfármaco, Qualigen, Zambon, UCB, Teva and KRKA and sponsorship from Zambon, TEVA and Abbvie for attending medical conferences.

Ines **Legarda** has received honoraria for educational presentations and advice service by

Abbvie, UCB Pharma, Zambon, Bial, and Teva.

Jorge **Hernández Vara** has received travel bursaries and educational grants from Abbvie and has received honoraria for educational presentations from Abbvie, Teva, Bial, Zambon, Italfarmaco, and Sanofi‐Genzyme.

Iria **Cabo**: has received honoraria for educational presentations and advice service by Abbvie, Zambon, and Bial.

Lydia **López Manzanares** compensated advisory services, consulting, research grant support, or speaker honoraria: AbbVie, Acorda, Bial, Intec Pharma, Italfarmaco, Pfizer, Roche, Teva, UCB, and Zambon.

Isabel **González Aramburu**: None.

Maria A. **Ávila Rivera** has received honoraria from Zambon, UCB Pharma, Qualigen, Bial, and Teva, and sponsorship from Zambon and Teva for attending conferences.

Víctor **Gómez Mayordomo** has received honoraria from Bial, Merz and Zambon for educational lectures.

Víctor Nogueira: None.

Julio **Dotor García‐Soto** compensated advisory services, consulting, research grant support, or speaker honoraria: Merck, Sanofi‐Genzyme, Allergan, Biogen, Roche, UCB and Novartis.

Carmen **Borrué**: None.

Berta **Solano Vila** has received honoraria for educational presentations and advice service by UCB, Zambon, Teva, Abbvie, Bial.

María **Álvarez Sauco** has received honoraria for educational presentations and advice service by Abbvie, UCB Pharma, Zambon, Bial, and Teva.

Lydia **Vela** has received honoraria for educational presentations and advice service by Abbvie, UCB Pharma, Lundbeck, KRKA, Zambon, Bial, and Teva.

Sonia **Escalante** has received honoraria for educational presentations and advice service by Abbvie, Zambon, and Bial.

Esther **Cubo**: Travel grants: Abbvie, Allergan, Boston; Lecturing honoraria: Abbvie, International Parkinson's disease Movement Disorder Society.

Zebenzui **Mendoza**: None.

Juan C. **Martínez Castrillo** has received research support from Lundbeck, Italfarmaco, Allergan, Zambon, Merz, and Abbvie. He has received speaking honoraria from AbbVie, Bial, Italfarmaco, Lundbeck, Krka, TEVA, UCB, Zambon, Allergan, Ipsen, and Merz.

Pilar **Sánchez Alonso** has received honoraria for educational presentations and advice service by Abbvie, UCB Pharma, Lundbeck, KRKA, Zambon, Bial, and Teva.

Maria G. **Alonso Losada** has received honoraria for educational presentations and advice service by Zambon and Bial.

Nuria **López Ariztegui** has received honoraria for educational presentations and advice service by Abbvie, Italfarmaco, Zambon, and Bial.

Itziar **Gastón** has received research support from Abbvie and Zambon and has served as a consultant for Abbvie, Exelts, and Zambon.

Jaime Kulisevsky: (1) Consulting fees: Roche, Zambon; (2) Stock / allotment: No; (3) Patent royalties / licensing fees: No; (4) Honoraria (e.g. lecture fees): Zambon, Teva, Bial, UCB; (5) Fees for promotional materials: No; (6) Research funding: Roche, Zambon, Ciberned; Instituto de SaludCarlos III; FundacióLa Maratóde TV3; (7) Scholarship from corporation: No; (8) Corporate laboratory funding: No; (9) Others (e.g. trips, travel, or gifts): No.

Manuel **Seijo** has received honoraria for educational services from KRKA, UCB, Zambon, Bial; travel grants from Daiichi and Roche.

Caridad **Valero** has received honoraria for educational services from Zambon, Abbvie and UCB.

Kurtis M. has received honoraria from Bial, the Spanish Neurology Society, and the International and Movement Disorders Society.

Ruben **Alonso Redondo**: None.

Maria Teresa **Buongiorno**: None.

Carlos **Ordás**: None.

Manuel **Menéndez‐González** has received consulting honoraria from Eisai Spain and research grants from by Instituto de Salud Carlos III (ISCIII), Fondo Europeo de Desarrollo Regional (FEDER), and Fundación Alimerka.

Darrian **McAfee**: None.

Pablo **Martinez‐Martin** has received honoraria from Bial for lecturing in course and from the Parkinson and Movement Disorder Society (MDS) for management of the COA International Program of the Society.

Pablo **Mir** has received honoraria from AbbVie, Abbott, Allergan, Bial, Merz, UCB, and Zambon and have received grants from the Spanish Ministry of Economy and Competitiveness [ PI16/01575] co‐founded by ISCIII (Subdirección General de Evaluación y Fomento de la Investigación) and by Fondo Europeo de Desarrollo Regional (FEDER), the Consejería de Economía, Innovación, Ciencia y Empleo de la Junta de Andalucía [CVI‐02526, CTS‐7685], the Consejería de Salud y Bienestar Social de la Junta de Andalucía [ PI‐0437‐2012, PI‐0471‐2013], the Sociedad Andaluza de Neurología, the Jacques and Gloria Gossweiler Foundation, the Fundación Alicia Koplowitz, the Fundación Mutua Madrileña.

### PEER REVIEW

The peer review history for this article is available at https://publons.com/publon/10.1002/brb3.3295.

## Data Availability

The data that support the findings of this study are available on request from the corresponding author. The data are not publicly available due to privacy or ethical restrictions.

## APPENDIX 1

### 1.1

COPPADIS STUDY GROUP

Adarmes AD, Almeria M, Alonso Losada MG, Alonso Cánovas A, Alonso Frech F, Alonso Redondo R, Álvarez I, Álvarez Sauco M, Aneiros Díaz A, Arnáiz S, Arribas S, Ascunce Vidondo A, Aguilar M, Ávila MA, Bernardo Lambrich N, Bejr‐Kasem H, Blázquez Estrada M, Botí M, Borrue C, Buongiorno MT, Cabello González C, Cabo López I, Caballol N, Cámara Lorenzo A, Canfield Medina H, Carrillo F, Carrillo Padilla FJ, Casas E, Catalán MJ, Clavero P, Cortina Fernández A, Cosgaya M, Cots Foraster A, Crespo Cuevas A, Cubo E, de Deus Fonticoba T, de Fábregues‐Boixar O, Díez‐Fairen M, Dotor García‐Soto J, Erro E, Escalante S, Estelrich Peyret E, Fernández Guillán N, Gámez P, Gallego M, García Caldentey J, García Campos C, García Díez C, García Moreno JM, Gastón I, Gómez Garre MP, Gómez Mayordomo V, González Aloy J, González‐Aramburu I, González Ardura J, González García B, González Palmás MJ, González Toledo GR, Golpe Díaz A, Grau Solá M, Guardia G, Hernández Vara J, Horta‐Barba A, Idoate Calderón D, Infante J, Jesús S, Kulisevsky J, Kurtis M, Labandeira C, Labrador MA, Lacruz F, Lage Castro M, Lastres Gómez S, Legarda I, López Ariztegui N, López Díaz LM, López Domínguez D, López Manzanares L, López Seoane B, Lucas del Pozo S, Macías Y, Mata M, Martí Andres G, Martí MJ, Martínez Castrillo JC, Martinez‐Martin P, McAfee D, Meitín MT, Mendoza Plasencia Z, Menéndez González M, Méndez del Barrio C, Mir P, Miranda Santiago J, Morales Casado MI, Moreno Diéguez A, Nogueira V, Novo Amado A, Novo Ponte S, Ordás C, Pagonabarraga J, Pareés I, Pascual‐Sedano B, Pastor P, Pérez Fuertes A, Pérez Noguera R, Planas‐Ballvé A, Planellas L, Prats MA, Prieto Jurczynska C, Puente V, Pueyo Morlans M, Puig Daví A, Redondo Rafales N, Rodríguez Méndez L, Rodríguez Pérez AB, Roldán F, Ruíz De Arcos M, Ruíz Martínez J, Sánchez Alonso P, Sánchez‐Carpintero M, Sánchez Díez G, Sánchez Rodríguez A, Santacruz P, Santos‐García D, Segundo Rodríguez JC, Seijo M, Sierra Peña M, Solano Vila B, Suárez Castro E, Tartari JP, Valero C, Vargas L, Vela L, Villanueva C, Vives B.

**Table:**

**Name (last Name, first Name)**	**Location**	**Role**	**Contribution**
Astrid Adarmes, Daniela	Hospital Universitario Virgen del Rocío, Sevilla, Spain	Site investigator	Evaluation of participants and/or data management
Almeria, Marta	Hospital Universitari Mutua de Terrassa, Terrassa, Barcelona, Spain	Site investigator	Neuropsychologist; evaluation of participants
Alonso Losada, Maria Gema	Hospital Álvaro Cunqueiro, Complejo Hospitalario Universitario de Vigo (CHUVI), Vigo, Spain	Site investigator/PI	Coordination at the center Evaluation of participants and/or data management
Alonso Cánovas, Araceli	Hospital Universitario Ramón y Cajal, Madrid, Spain	Site investigator	Evaluation of participants and/or data management
Alonso Frech, Fernando	Hospital Universitario Clínico San Carlos, Madrid, Spain	Site investigator	Evaluation of participants and/or data management
Alonso Redondo, Ruben	Hospital Universitario Lucus Augusti (HULA), Lugo, Spain	Site investigator/PI	Coordination at the center Evaluation of participants and/or data management
Aneiros Díaz, Ángel	Complejo Hospitalario Universitario de Ferrol (CHUF), Ferrol, A Coruña, Spain	Site investigator/PI	Coordination at the center Evaluation of participants and/or data management
Álvarez, Ignacio	Hospital Universitari Mutua de Terrassa, Terrassa, Barcelona, Spain	Site investigator	Evaluation of participants and/or data management
Álvarez Sauco, María	Hospital General Universitario de Elche, Elche, Spain	Site investigator/PI	Coordination at the center Evaluation of participants and/or data management
Arnáiz, Sandra	Complejo Asistencial Universitario de Burgos, Burgos, Spain	Site investigator	Evaluation of participants and/or data management
Arribas, Sonia	Hospital Universitari Mutua de Terrassa, Terrassa, Barcelona, Spain	Site investigator	Neuropsychologist; evaluation of participants
Ascunce Vidondo, Arancha	Complejo Hospitalario de Navarra, Pamplona, Spain	Site investigator	Evaluation of participants and/or data management
Aguilar, Miquel	Hospital Universitari Mutua de Terrassa, Terrassa, Barcelona, Spain	Site investigator	Evaluation of participants and/or data management
Ávila Rivera, Maria Asunción	Consorci Sanitari Integral, Hospital General de L´Hospitalet, L´Hospitalet de Llobregat, Barcelona, Spain	Site investigator/PI	Coordination at the center Evaluation of participants and/or data management
Bernardo Lambrich, Noemí	Hospital de Tortosa Verge de la Cinta (HTVC), Tortosa, Tarragona, Spain	Site investigator	Evaluation of participants and/or data management
Bejr‐Kasem, Helena	Hospital de Sant Pau, Barcelona, Spain	Site investigator	Evaluation of participants and/or data management
Blázquez Estrada, Marta	Hospital Universitario Central de Asturias, Oviedo, Spain	Site investigator	Evaluation of participants and/or data management
Botí González, Maria Ángeles	Hospital Universitari Mutua de Terrassa, Terrassa, Barcelona, Spain	Site investigator	Neuropsychologist; evaluation of participants
Borrué, Carmen	Hospital Infanta Sofía, Madrid, Spain	Site investigator/PI	Coordination at the center Evaluation of participants and/or data management
Buongiorno, Maria Teresa	Hospital Universitari Mutua de Terrassa, Terrassa, Barcelona, Spain	Site investigator	Nurse study coordinator
Cabello González, Carolina	Complejo Hospitalario de Navarra, Pamplona, Spain	Site investigator	Scheduling of evaluations
Cabo López, Iria	Complejo Hospitalario Universitario de Pontevedra (CHOP), Pontevedra, Spain	Site investigator/PI	Coordination at the center Evaluation of participants and/or data management
Caballol, Nuria	Consorci Sanitari Integral, Hospital Moisés Broggi, Sant Joan Despí, Barcelona, Spain	Site investigator/PI	Coordination at the center Evaluation of participants and/or data management
Cámara Lorenzo, Ana	Hospital Clínic de Barcelona, Barcelona, Spain	Site investigator	Nurse study coordinator
Canfield Medina, Héctor	Complejo Hospitalario Universitario de Ferrol (CHUF), Ferrol, A Coruña, Spain	Site investigator	Evaluation of participants and/or data management
Carrillo, Fátima	Hospital Universitario Virgen del Rocío, Sevilla, Spain	Site investigator	Evaluation of participants and/or data management
Carrillo Padilla, Francisco José	Hospital Universitario de Canarias, San Cristóbal de la Laguna, Santa Cruz de Tenerife, Spain	Site investigator/PI	Coordination at the center Evaluation of participants and/or data management
Casas, Elena	Complejo Asistencial Universitario de Burgos, Burgos, Spain	Site investigator	Evaluation of participants and/or data management
Catalán, Maria José	Hospital Universitario Clínico San Carlos, Madrid, Spain	Site investigator/PI	Coordination at the center Evaluation of participants and/or data management
Clavero, Pedro	Complejo Hospitalario de Navarra, Pamplona, Spain	Site investigator	Evaluation of participants and/or data management
Cortina Fernández, A	Complejo Hospitalario Universitario de Ferrol (CHUF), Ferrol, A Coruña, Spain	Site investigator	Coordination of blood extractions
Cosgaya, Marina	Hospital Clínic de Barcelona, Barcelona, Spain	Site investigator	Evaluation of participants and/or data management
Cots Foraster, Anna	Institut d'Assistència Sanitària (IAS)—Instituí Cátala de la Salud. Girona, Spain	Site investigator	Evaluation of participants and/or data management
Crespo Cuevas, Ane	Hospital del Mar, Barcelona, Spain	Site investigator	Evaluation of participants and/or data management
Cubo, Esther	Complejo Asistencial Universitario de Burgos, Burgos, Spain	Site investigator/PI	Coordination at the center Evaluation of participants and/or data management
De Deus Fonticoba, Teresa	Complejo Hospitalario Universitario de Ferrol (CHUF), Ferrol, A Coruña, Spain	Site investigator	Nurse study coordinator Evaluation of participants and/or data management
De Fábregues‐Boixar, Oriol	Hospital Universitario Vall d´Hebron, Barcelona, Spain	Site investigator/PI	Coordination at the center Evaluation of participants and/or data management
Díez Fairen, M	Hospital Universitari Mutua de Terrassa, Terrassa, Barcelona, Spain	Site investigator	Evaluation of participants and/or data management
Dotor García‐Soto, Julio	Hospital Universitario Virgen Macarena, Sevilla, Spain	Site investigator/PI	Evaluation of participants and/or data management
Erro, Elena	Complejo Hospitalario de Navarra, Pamplona, Spain	Site investigator	Evaluation of participants and/or data management
Escalante, Sonia	Hospital de Tortosa Verge de la Cinta (HTVC), Tortosa, Tarragona, Spain	Site investigator/PI	Coordination at the center Evaluation of participants and/or data management
Estelrich Peyret, Elena	Institut d'Assistència Sanitària (IAS)—Instituí Cátala de la Salud. Girona, Spain	Site investigator	Evaluation of participants and/or data management
Fernández Guillán, Noelia	Complejo Hospitalario Universitario de Ferrol (CHUF), Ferrol, A Coruña, Spain	Site investigator	Neuroimaging studies
Gámez, Pedro	Complejo Asistencial Universitario de Burgos, Burgos, Spain	Site investigator	Evaluation of participants and/or data management
Gallego, Mercedes	Hospital La Princesa, Madrid, Spain	Site investigator	Evaluation of participants and/or data management
García Caldentey, Juan	Centro Neurológico Oms 42, Palma de Mallorca, Spain	Site investigator/PI	Coordination at the center Evaluation of participants and/or data management
García Campos, Cristina	Hospital Universitario Virgen Macarena, Sevilla, Spain	Site investigator	Evaluation of participants and/or data management
García Díez, Cristina	Complejo Hospitalario Universitario de Pontevedra (CHOP), Pontevedra, Spain	Site investigator (from MAY/22)	neuropsychologist; evaluation of participants
García Moreno, Jose Manuel	Hospital Universitario Virgen Macarena, Sevilla, Spain	Site investigator/PI (until MAR/21)	Coordination at the center Evaluation of participants and/or data management
Gastón, Itziar	Complejo Hospitalario de Navarra, Pamplona, Spain	Site investigator/PI	Coordination at the center Evaluation of participants and/or data management
Gómez Garre, María del Pilar	Hospital Universitario Virgen del Rocío, Sevilla, Spain	Site investigator	Genetic studies coordination
Gómez Mayordomo, Víctor	Hospital Clínico San Carlos, Madrid, Spain	Site investigator	Evaluation of participants and/or data management
González Aloy, Javier	Institut d'Assistència Sanitària (IAS)—Instituí Cátala de la Salud. Girona, Spain	Site investigator	Evaluation of participants and/or data management
González Aramburu, Isabel	Hospital Universitario Marqués de Valdecilla, Santander, Spain	Site investigator	Evaluation of participants and/or data management
González Ardura, Jessica	Hospital Universitario Lucus Augusti (HULA), Lugo, Spain	Site investigator/PI (until FEB/21)	Evaluation of participants and/or data management
González García, Beatriz	Hospital La Princesa, Madrid, Spain	Site investigator	Nurse study coordinator
González Palmás, Maria Josefa	Complejo Hospitalario Universitario de Pontevedra (CHOP), Pontevedra, Spain	Site investigator	Evaluation of participants and/or data management
González Toledo, Gabriel Ricardo	Hospital Universitario de Canarias, San Cristóbal de la Laguna, Santa Cruz de Tenerife, Spain	Site investigator	Evaluation of participants and/or data management
Golpe Díaz, Ana	Complejo Hospitalario Universitario de Ferrol (CHUF), Ferrol, A Coruña, Spain	Site investigator	Laboratory analysis coordination
Grau Solá, Mireia	Consorci Sanitari Integral, Hospital Moisés Broggi, Sant Joan Despí, Barcelona, Spain	Site investigator	Evaluation of participants and/or data management
Guardia, Gemma	Hospital Universitari Mutua de Terrassa, Terrassa, Barcelona, Spain	Site investigator	Evaluation of participants and/or data management
Hernández Vara, Jorge	Hospital Universitario Vall d´Hebron, Barcelona, Spain	Site investigator/PI	Coordination at the center Evaluation of participants and/or data management
Horta Barba, Andrea	Hospital de Sant Pau, Barcelona, Spain	Site investigator	Neuropsychologist; evaluation of participants
Idoate Calderón, Daniel	Complejo Hospitalario Universitario de Pontevedra (CHOP), Pontevedra, Spain	Site investigaor (until MAY/22)	neuropsychologist; evaluation of participants
Infante, Jon	Hospital Universitario Marqués de Valdecilla, Santander, Spain	Site investigator/PI	Coordination at the center Evaluation of participants and/or data management
Jesús, Silvia	Hospital Universitario Virgen del Rocío, Sevilla, Spain	Site investigator	Evaluation of participants and/or data management
Kulisevsky, Jaime	Hospital de Sant Pau, Barcelona, Spain	Site investigator/PI	Coordination at the center Evaluation of participants and/or data management
Kurtis, Mónica	Hospital Ruber Internacional, Madrid, Spain	Site investigator/PI	Coordination at the center Evaluation of participants and/or data management
Labandeira, Carmen	Hospital Álvaro Cunqueiro, Complejo Hospitalario Universitario de Vigo (CHUVI), Vigo, Spain	Site investigator	Evaluation of participants and/or data management
Labrador Espinosa, Miguel Ángel	Hospital Universitario Virgen del Rocío, Sevilla, Spain	Site investigator	Neuroimaging data analysis
Lacruz, Francisco	Complejo Hospitalario de Navarra, Pamplona, Spain	Site investigator	Evaluation of participants and/or data management
Lage Castro, Melva	Complejo Hospitalario Universitario de Pontevedra (CHOP), Pontevedra, Spain	Site investigator	Evaluation of participants and/or data management
Lastres Gómez, Sonia	Complejo Hospitalario Universitario de Pontevedra (CHOP), Pontevedra, Spain	Site investigator	Neuropsychologist; evaluation of participants
Legarda, Inés	Hospital Universitario Son Espases, Palma de Mallorca, Spain	Site investigator/PI	Coordination at the center Evaluation of participants and/or data management
López Ariztegui, Nuria	Complejo Hospitalario de Toledo, Toledo, Spain	Site investigator/PI	Evaluation of participants and/or data management
López Díaz, Luis Manuel	Hospital Da Costa de Burela, Lugo, Spain	Site investigator	Evaluation of participants and/or data management
López Domínguez, Daniel	Institut d'Assistència Sanitària (IAS)—Instituí Cátala de la Salud. Girona, Spain	Site investigator	Evaluation of participants and/or data management
López Manzanares, Lydia	Hospital La Princesa, Madrid, Spain	Site investigator/PI	Coordination at the center Evaluation of participants and/or data management
López Seoane, Balbino	Complejo Hospitalario Universitario de Ferrol (CHUF), Ferrol, A Coruña, Spain	Site investigator	Neuroimaging studies
Lucas del Pozo, Sara	Hospital Universitario Vall d´Hebron, Barcelona, Spain	Site investigator	Evaluation of participants and/or data management
Macías, Yolanda	Fundación Hospital de Alcorcón, Madrid, Spain	Site investigator	Evaluation of participants and/or data management
Mata, Marina	Hospital Infanta Sofía, Madrid, Spain	Site investigator	Evaluation of participants and/or data management
Martí Andres, Gloria	Hospital Universitario Vall d´Hebron, Barcelona, Spain	Site investigator	Evaluation of participants and/or data management
Martí, Maria José	Hospital Clínic de Barcelona, Barcelona, Spain	Site investigator/PI	Coordination at the center Evaluation of participants and/or data management
Martínez Castrillo, Juan Carlos	Hospital Universitario Ramón y Cajal, Madrid, Spain	Site investigator/PI	Coordination at the center Evaluation of participants and/or data management
Martinez‐Martin, Pablo	Centro Nacional de Epidemiología y CIBERNED, Instituto de Salud Carlos III. Madrid	Collaborator in statistical and methods analysis	Methods and statistical reviewer
McAfee, Darrian	University of Pennsylvania, Philadelphia	Collaborator in english style	English style reviewer
Meitín, Maria Teresa	Hospital Da Costa de Burela, Lugo, Spain	Site investigator	Evaluation of participants and/or data management
Menéndez González, Manuel	Hospital Universitario Central de Asturias, Oviedo, Spain	Site investigator/PI	Coordination at the center Evaluation of participants and/or data management
Méndez del Barrio, Carlota	Hospital Universitario Virgen del Rocío, Sevilla, Spain	Site investigator	Evaluation of participants and/or data management
Mendoza Plasencia, Zebenzui	Hospital Universitario de Canarias, San Cristóbal de la Laguna, Santa Cruz de Tenerife, Spain	Site investigator	Evaluation of participants and/or data management
Mir, Pablo	Hospital Universitario Virgen del Rocío, Sevilla, Spain	Site investigator/PI	Coordination at the center Evaluation of participants and/or data management
Miranda Santiago, Javier	Complejo Asistencial Universitario de Burgos, Burgos, Spain	Site investigator	Evaluation of participants and/or data management
Morales Casado, Maria Isabel	Complejo Hospitalario de Toledo, Toledo, Spain	Site investigator	Evaluation of participants and/or data management
Moreno Diéguez, Antonio	Complejo Hospitalario Universitario de Ferrol (CHUF), Ferrol, A Coruña, Spain	Site investigator	Neuroimaging studies
Nogueira, Víctor	Hospital Da Costa de Burela, Lugo, Spain	Site investigator/PI	Coordination at the center Evaluation of participants and/or data management
Novo Amado, Alba	Complejo Hospitalario Universitario de Ferrol (CHUF), Ferrol, A Coruña, Spain	Site investigator	Neuroimaging studies
Novo Ponte, Sabela	Hospital Universitario Puerta de Hierro, Madrid, Spain	Site investigator	Evaluation of participants and/or data management
Ordás, Carlos	Hospital Rey Juan Carlos, Madrid, Spain, Madrid, Spain	Site Investigator	Evaluation of participants and/or data management
Pagonabarraga, Javier	Hospital de Sant Pau, Barcelona, Spain	Site investigator	Evaluation of participants and/or data management
Pareés, Isabel	Hospital Ruber Internacional, Madrid, Spain	Site investigator	Evaluation of participants and/or data management
Pascual‐Sedano, Berta	Hospital de Sant Pau, Barcelona, Spain	Site Investigator	Evaluation of participants and/or data management
Pastor, Pau	Hospital Universitari Mutua de Terrassa, Terrassa, Barcelona, Spain	Site investigator	Evaluation of participants and/or data management
Pérez Fuertes, Aída	Complejo Hospitalario Universitario de Ferrol (CHUF), Ferrol, A Coruña, Spain	Site investigator	Blood analysis
Pérez Noguera, Rafael	Hospital Universitario Virgen Macarena, Sevilla, Spain	Site investigator	Evaluation of participants and/or data management
Planas‐Ballvé, Ana	Consorci Sanitari Integral, Hospital Moisés Broggi, Sant Joan Despí, Barcelona, Spain	Site investigator	Evaluation of participants and/or data management
Planellas, Lluís	Hospital Clínic de Barcelona, Barcelona, Spain	Site investigator (until DEC/19)	Evaluation of participants and/or data management
Prats, Marian Ángeles	Institut d'Assistència Sanitària (IAS)—Instituí Cátala de la Salud. Girona, Spain	Site investigator	Evaluation of participants and/or data management
Prieto Jurczynska, Cristina	Hospital Rey Juan Carlos, Madrid, Spain, Madrid, Spain	Site investigator/PI	Coordination at the center Evaluation of participants and/or data management
Puente, Víctor	Hospital del Mar, Barcelona, Spain	Site investigator/PI	Coordination at the center Evaluation of participants and/or data management
Pueyo Morlans, Mercedes	Hospital Universitario de Canarias, San Cristóbal de la Laguna, Santa Cruz de Tenerife, Spain	Site investigator	Evaluation of participants and/or data management
Puig Daví, Arnau	Hospital de Sant Pau, Barcelona, Spain	Site einvestigator	Evaluation of participants and/or data management
Redondo, Nuria	Hospital La Princesa, Madrid, Spain	Site Investigator	Evaluation of participants and/or data management
Rodríguez Méndez, Luisa	Complejo Hospitalario Universitario de Ferrol (CHUF), Ferrol, A Coruña, Spain	Site investigator	Blood analysis
Rodríguez Pérez, Amparo Belén	Hospital General Universitario de Elche, Elche, Spain	Site investigator	Evaluation of participants and/or data management
Roldán, Florinda	Hospital Universitario Virgen del Rocío, Sevilla, Spain	Site investigator	Neuroimaging studies
Ruíz de Arcos, María	Hospital Universitario Virgen Macarena, Sevilla, Spain	Site investigator	Evaluation of participants and/or data management
Ruíz Martínez, Javier	Hospital Universitario Donostia, San Sebastián, Spain	Site investigator	Evaluation of participants and/or data management
Sánchez Alonso, Pilar	Hospital Universitario Puerta de Hierro, Madrid, Spain	Site investigator	Evaluation of participants and/or data management
Sánchez‐Carpintero, Macarena	Complejo Hospitalario Universitario de Ferrol (CHUF), Ferrol, A Coruña, Spain	Site investigator	Neuroimaging studies
Sánchez Díez, Gema	Hospital Universitario Ramón y Cajal, Madrid, Spain	Site investigator	Evaluation of participants and/or data management
Sánchez Rodríguez, Antonio	Hospital Universitario Marqués de Valdecilla, Santander, Spain	Site investigator	Evaluation of participants and/or data management
Santacruz, Pilar	Hospital Clínic de Barcelona, Barcelona, Spain	Site investigator	Evaluation of participants and/or data management
Santos‐García, Diego	CHUAC, Complejo Hospitalario Universitario de A Coruña	Coordinator of the Project	Coordination of the COPPADIS‐2015
Segundo Rodríguez, José Clemente	Complejo Hospitalario de Toledo, Toledo, Spain	Site investigator	Evaluation of participants and/or data management
Seijo, Manuel	Complejo Hospitalario Universitario de Pontevedra (CHOP), Pontevedra, Spain	Site investigator/PI	Coordination at the center Evaluation of participants and/or data management
Sierra, María	Hospital Universitario Marqués de Valdecilla, Santander, Spain	Site investigator	Evaluation of participants and/or data management
Solano, Berta	Institut d'Assistència Sanitària (IAS)—Instituí Cátala de la Salud. Girona, Spain	Site investigator/PI	Coordination at the center Evaluation of participants and/or data management
Suárez Castro, Ester	Complejo Hospitalario Universitario de Ferrol (CHUF), Ferrol, A Coruña, Spain	Site investigator	Evaluation of participants and/or data management
Tartari, Juan Pablo	Hospital Universitari Mutua de Terrassa, Terrassa, Barcelona, Spain	Site investigator	Evaluation of participants and/or data management
Valero, Caridad	Hospital Arnau de Vilanova, Valencia, Spain	Site investigator	Evaluation of participants and/or data management
Vargas, Laura	Hospital Universitario Virgen del Rocío, Sevilla, Spain	Site investigator	Evaluation of participants and/or data management
Vela, Lydia	Fundación Hospital de Alcorcón, Madrid, Spain	Site investigator/PI	Coordination at the center Evaluation of participants and/or data management
Villanueva, Clara	Hospital Universitario Clínico San Carlos, Madrid, Spain	Site investigator	Evaluation of participants and/or data management
Vives, Bárbara	Hospital Universitario Son Espases, Palma de Mallorca, Spain	Site investigator	Evaluation of participants and/or data management
